# Data mining of key genes expression in hepatocellular carcinoma: novel potential biomarkers of diagnosis prognosis or progression

**DOI:** 10.1007/s10585-022-10164-9

**Published:** 2022-04-16

**Authors:** Manuela Cabiati, Melania Gaggini, Paolo De Simone, Silvia Del Ry

**Affiliations:** 1 Biochemistry and Molecular Biology Lab, Institute of Clinical Physiology – National Research Council CNR, Via Giuseppe Moruzzi 1, 56124 Pisa, Italy; 2 Hepatobiliary Surgery and Liver Transplantation, University of Pisa Medical School Hospital, Pisa, Italy

**Keywords:** Data mining, HCC, Key genes, Osteopontin

## Abstract

Hepatocellular carcinoma (HCC) is one of the main cancer-related causes of death worldwide. The study aimed to perform a data mining analysis of the expression and regulatory role of key genes in HCC to reveal novel potential biomarkers of diagnosis prognosis, or progression since their availability is still almost lacking. Starting from data of our cohort of patients (HCV-positive HCC pts undergoing liver transplantation (LR, n = 10) and donors (LD, n = 14), deeply analyzed previously, in which apelin, osteopontin, osteoprotegerin, NOTCH-1, CASP-3, Bcl-2, BAX, PTX3, and NPTX2 were analyzed, we applied statistical analysis and in-silico tools (Gene Expression Profiling Interactive Analysis, HCCDB database and GeneMania, UALCAN) to screen and identify the key genes. Firstly, we performed a stepwise regression analysis using our mRNA-datasets which revealed that higher expression levels of apelin and osteopontin were positively associated with the HCC and identified that the most consistently differentially expressed gene across multiple HCC expression datasets was only OPN. This comprehensive strategy of data mining evidenced that OPN might have a potential function as an important tumor marker-driven oncogenesis being associated with poor prognosis of HCC patients.

**Supplementary Information:**

The online version contains supplementary material available at 10.1007/s10585-022-10164-9.

## Introduction

Primary liver cancer includes hepatocellular carcinoma (HCC), intrahepatic cholangiocarcinoma, andother rare types, with the exclusion of secondary liver cancer. HCC is one of the major cancer deaths representing the third leading cause worldwide [1] and the foremost cause of liver transplant. The incidence of HCC is growing in most countries and it accounts for 79% of the total primary liver cancers while cholangiocarcinoma and adenocarcinoma only 6% and 4%, respectively [2]. The Global Cancer Observatory database (GLOBOCAN), an interactive web-based platform presenting global cancer statistics, shows that in Italy HCC ranks at 13° place representing about 3% of all new cancer diagnoses. In 2018, HCC was estimated in Italy and about 12,800 diagnoses of new cases of HCC with a male:female ratio of approximately 2.2:1 were reported. The 5-year survival of patients with HCC is 20% while it further halves after 10 years from the first diagnosis (10%) even though no significant differences are observed at a national level concerning other types of neoplasms with a severe prognosis [3, 4]. The etiology of HCC is likely multifactorial and it is plausible that HCC might results from alterations in lipid metabolism, cell lipotoxicity, insulin resistance, and oxidative stress since subjects with non-alcoholic fatty liver disease (NAFLD) are at high risk of development and progression of HCC [5, 6]. At present, the non-alcoholic steatohepatitis (NASH) and obesity represent, at least in developed countries, the most important cause of HCC, and probably in the next future they could become the main cause for developing HCC [7] as well as alcohol abuse, that strongly interacts with other causes of liver damage [e.g. hepatitis B (HBV) and C (HCV)], worsening the progression of the disease and the development of HCC [8]. Moreover, HBV and HCV represent the main risk factors for HCC and are responsible for approximately 85% of HCC cases worldwide [9–12].

The main benefit of a cancer screening program is having a curative therapeutic option available. In the HCC setting, this goal is achieved when the diagnosis of a small HCC (smaller than 2 cm) is established, possibly single and within the Milan criteria and therefore treatable with curative intent therapies (transplantation, resection, ablation).

It is well known that HCC patients within the Milan criteria (solitary tumor ≤ 5 cm or ≤ 3 tumor, each < 3 cm) could undergo liver transplantation with excellent results. However, there is a growing tendency to enlarge inclusion criteria since the Milan criteria are nowadays considered too restrictive and may exclude patients who would benefit from liver transplantation. For this reason, the Milan criteria have been included in the Barcelona-Clínic Liver Cancer (BCLC) pre-transplant staging, in the American Association for the Study of Liver Diseases (AASLD), and the European Association for the Study of the Liver-European Organisation for Research and Treatment of Cancer (EASL-EORTC) practice guidelines [13]. The BCLC staging classification links the stage of the disease to a specific treatment strategy incorporating prognosis estimation and potential treatment advancements in a single unified proposal to select patients that could benefit from curative therapies [14]. The addition of the periodic assay of alpha-fetoprotein to ultrasound surveillance remains poorly understood, as it does not substantially increase the recognition rate of the early HCC stage [15, 16].

Thus, to date, early diagnosis of HCC is still very difficult and an evidence-based multidisciplinary and multi-axis approach to better characterize this pathology is still lacking even if the HCC scenario is continuously and rapidly evolving in terms of etiology and clinical presentation [2]. Moreover, metabolomics and transcriptomics have been effectively used to identify non-invasive biomarkers of organ metabolism. Of particular interest are the molecules that are markers of metabolic, apoptotic, inflammatory, and signaling pathways [17]. Recent studies by our group have analyzed a broad spectrum of biomarkers involved in HCC [18–22] within a larger flagship project coordinated by the National Research Council (INTEROMICS FLAGSHIP PROJECT supported by the Italian Ministry of Education, University and Research, MUR, and, call 2015 and 2018). In particular, the behavior of the apelinergic axis (APLN), Osteopontin (OPN or SPP1), osteoprotegerin (OPG/TNFRSF11B), apoptotic markers Bcl-2, BCLXL, NOTCH-1 ad inflammatory cytokines (IL-6, TNF-α, PTX3, and NPTX2) was deeply analyzed in patients submitted to primary, whole-size, liver transplantation and in deceased donors, providing new evidence on the expression of these biomarkers in HCC, as reported in our recent works [19–22], filling, at least in part, the gaps present in HCC literature about their trend. As observed in other types of malignancies, these new data highlight their strong action in tumor progression and aggressiveness also during HCC [23–29].

Starting from the results obtained by our studies [19–22] we aimed to perform a data mining analyses of the expression and regulatory role of key genes in HCC to reveal novel potential biomarkers of diagnosis prognosis, or progression. In particular, an in-silico analysis through Gene Expression Profiling Interactive Analysis (GEPIA), HCCDB database, GeneMania, and UALCAN was carried out to screen and identify the key genes.

## Material and methods

### Patients selection criteria

The present report is a prospective, single-Center study in which the patients were deeply characterized by a bio-humoral and biomolecular point of view as reported in previous studies of ours [18–22]. The cohort of patients enrolled was submitted to primary, whole-size, liver transplantation at the Liver Transplantation Unit of the University of Pisa where the patients with HCC were admitted for surgery after providing written informed consent. As controls, deceased donors were studied.

The current study population included twenty-eight adult subjects: 14 subjects with HCV-related HCC undergoing liver transplantation (liver recipients, LR, age 59.4 ± 1.8 years) and 14 donors (liver donors, LD, age 62.1 ± 17.3 years). Four patients with HCV- related HCC resulted diabetic, and for consistency of results were excluded from the analysis. The inclusion and exclusion criteria as well as the MELD score at transplant were carefully considered as detailed in our previous works [18–22]. In particular, the MELD score was chosen as a criterion for organ allocation in patients undergoing liver transplantation with increased risk of mortality on the waiting list, thus LR were divided in those with MELD score < 9 (about 2% mortality following literature data) and those with a MELD score between 10 and 13 (experienced a 6% mortality). Due to the criteria used for the allocation of donor’s livers, the subjects enrolled in the study not exceeded MELD score of 13.

All protocols of this study were approved by the Institutional Ethics Committee, according to the Code of Ethics of the World Medical Association (Declaration of Helsinki) for experiments involving human subjects.

### Transcriptional analysis: real-time PCR experiments

Hepatic samples handling, RNA extraction, quality, and cDNA synthesis were carried out as previously reported [19–22]. Briefly, the tissue samples were collected at the time of liver explant from the donor liver graft and the recipient’s liver. Total RNA was extracted following the manufacturer's instructions (Rneasy Mini kit Qiagen S.p.A, Italy, MI) and cDNA was synthesized with iScript cDNA Synthesis kit (Bio-Rad, Hercules, CA, USA) [19–22].

In Table 1 are listed the primer pairs, synthesized by Sigma-Aldrich (St. Louis, MO, USA), of both key genes, used for data mining analysis, and the three reference genes, previously selected and used for the normalization of Real-Time PCR data [19–22].

**Table 1:** Primer sequence details of the analyzed gene

Genes	Primer sequence	GenBank, accession n.	Length (pb)	Annealing Temp (°C)	Efficiency (%)	R^2^
eEF1a	**F:** CTTTGGGTCGCTTTGCTGTT	NM_001402	183	60	101.7	0.998
	**R:** CCGTTCTTCCACCACTGATT					
PPIA	**F:** CTTGGGCCGCGTCTCCTTCG	NM_021130	285	60	103.4	0.998
	**R:** TTGGGAACCGTTTGTGTTTGGGGC					
TPT1	**F:** AAATGTTAACAAATGTGGCAATT	NM_003295	164	60	105	0.999
	**R:** AACAATGCCTCCACTCCAAA					
OPN	**F:** AATGATGAGAGCAATGAG **R:** GTCTACAACCAGCATATC	NM_001040058	114	60	103	0.999
OPG	**F:** GACGAAGAAACCTCTCATCA **R:** GCTGTCTGTGTAGTAGTGG	U94332	126	60	105	0.998
APLN	**F:** CTTCTCACCTGCCTGCTT	NM_ 017413	88	60	97.9	0.991
	**R:** ATGGACTGGACGGATTCTTG					
Bcl-2	**F:** CCGACCACTAATTGCCAAG	NM_000633	121	58	99.6	0.995
	**R:** TTCCATCCGTCTGCTCTT					
BAX	**F:** ACCAGGGTGGTTGGGTGAGACTC **R:** TCCAGGGAGGGCAGAAGGCACTA	AY217036.1	289	58	101.2	0.994
NOTCH-1	**F:** AGAACTGTGAGGAAAATATCG **R:** TACTGACCTGTCCACTCT	AF308602	118	60	105	0.990
CASP-3	**F:** CTGTAACTTGAGAGTAGATGGT **R:** ATGGAGAAATGGGCTGTAG	NM_032991	110	60	104.8	0.997
PTX3	**F:** AATGCTGTGTCTCTGTCA **R:** ACATACCAATAACAATGAACAATG	NM_002852	175	62	104.7	0.998
NPTX2	**F:** AAAGGAGAAGGGCTGTGAT **R:** GGCACCAGATGAGAAGAGA	NM_002523	116	60	97	0.996

*eEF1a* Eukaryotic translation elongation factor 1 alpha 1, *PPIA* peptidylpropyl isomerase A [cyclophilin A], *TPT1* tumor protein, translationally controlled 1, *OPN* osteopontin or secreted phosphoprotein 1 (SPP1), *OPG* osteoprotegerin or tumor necrosis factor receptor superfamily member 11B (TNFRSF11B), *APLN* apelin, *Bcl2* beta-cell leukemia 2, *BAX* bcl-2-like protein 4, *NOTCH-1* neurogenic locus notch homolog protein 1, *CASP-3* caspase 3, *PTX3* pentraxin 3; *NPTX2* neural pentraxin 2

Real-Time PCR amplification and analysis were conducted in a Bio-Rad CFX96™ Real-Time System (Bio-Rad Laboratories Inc., Hercules, CA, USA) with a sybrgreen assay.

### Statistical analysis

A descriptive analysis to describe the baseline characteristics (age, gender, diagnosis, HCC genotype, liver disease severity, and biochemical parameters) of HCC patients and donors was performed.

In an effort to provide greater transparency of our results between research laboratories, this study was carried out conforming to the “Minimum Information for publication of Quantitative Real-Time PCR Experiments (MIQE)” [30], as listed in Table 1. The GeNorm normalization strategy was used to assess the expression stability of each candidate reference gene, to determine the ideal number of genes required for normalization, and to calculate individual normalization factors for each sample based on the expression levels of the best reference genes. The average C_t_ values obtained from each duplicate were converted to a relative quantity (∆C_t_) and analyzed with GeNorm algorithm. The geometric mean of the 3 most stably expressed genes (eEF1a, PPIA, TPT1), as previously reported [19–22], was applied to normalize Real-Time PCR data using the ΔΔC_t_ algorithm for relative quantification (CFX-96 Real-Time PCR detection systems, Bio-Rad Laboratories Inc., Hercules, CA, USA).

The Kolmogorov–Smirnov test of normality was used to verify whether the distribution of variables followed a Gaussian pattern. To analyze the control group versus patient data, Mann–Whitney for numerical factors were applied. Natural logarithmic transformation was applied to parameters that were not normally distributed in the study population before entering the statistical analysis. To investigate the association between the expression of our key genes in HCC, a multi-biomarker model was built and stepwise regression analysis (logistic model) was used to select the best performing variables in the predictive models.

The statistical analysis was performed using the statistical software Statview 5.0 software released for Windows Statistical (SAS Institute, Inc., Cary, NC, USA).

### Data mining

#### Genetic interaction analysis (GeneMANIA)

GeneMANIA (http://genemania.org/) is a web interface that uses large sets of functional association data to identify single genes related to a set of input genes [31] making fast and efficient function predictions. GeneMANIA was used to build a biological network and in our model, we examined “coexpression” “colocalization” “pathway” and “genetic interactions” of our key genes.

#### Gene Expression Profiling Interactive Analysis (GEPIA)

The Gene Expression Profiling Interactive Analysis (GEPIA, http://gepia.cancer-pku.cn/) is a web-based tool to deliver fast and customizable functionalities to complement the existing tools. The GEPIA database integrates TCGA data and GTEx normal tissue data (9736 tumors vs 8587 normal samples), and these data were used to analyze tumor/normal differential expression profiles, expression distribution, pathological staging, survival analysis results, the similarity between genes, gene expression correlation, etc. [32]. With the GEPIA database, the prognostic values of our key genes including overall survival (OS) and Disease-free survival (RFS) were evaluated in liver cancer patients as well as the expression profiles (DIY) according to HCC disease based on the TCGA clinical annotation. The expression data used to build the box plot are first log_2_(TPM + 1) transformed for differential analysis and the log_2_FC is defined as median (Tumor)—median (Normal) (where TPM corresponds to transcript per million and FC to fold-change threshold). Genes with higher |log_2_FC| values and lower q values (where q is the adjusted p-value) than pre-set thresholds are considered differentially expressed genes. The absolute value of Log_2_ Fold-change > 1.5 and p < 0.05 were set as the thresholds of gene upregulation. With the GEPIA database, a multiple gene analysis comparison was also performed. This feature provides expression matrix plots based on a given gene list. The density of color in each block represents the median expression value of a gene in a given tissue, normalized by the maximum median expression value across all blocks.

#### Integrative molecular database of hepatocellular carcinoma (HCCDB)

HCCDB is a web-based database, aiming at providing a one-stop resource for gene expression atlas in HCC (http://lifeome.net/database/hccdb/home.html).

Fifteen public dataset sets (13 GEO microarray datasets and two RNA-Seq datasets, TCGA-LICH and ICGC LIRI-JP) of HCC gene expression were archived in the HCCDB database including 3917 samples [33]. All GEO datasets are available from the GEO repository (https://www.ncbi.nlm.nih.gov/gds).

HCCDB database was used to confirm whether our genes were significantly differentially expressed in HCC: genes detected in at least 8 datasets and significantly differentially expressed in at least half of the datasets containing these genes are identified as uniformly differentially expressed. The dataset accession numbers are: GSE22058 (HCCDB1), GSE25097 (HCCDB3), GSE36376 (HCCDB4), GSE14520 (GPL3721 Subset, HCCDB6), GSE10143 (HCCDB7), GSE9843 (HCCDB8), GSE19977 (HCCDB9), GSE46444 (HCCDB11), GSE54236 (HCCDB12), GSE63898 (HCCDB13), GSE43619 (HCCDB14), TCGA-LIHC (HCCDB15, https://portal.gdc.cancer.gov/projects/TCGA-LIHC) GSE64041 (HCCDB16) and GSE76427 (HCCDB17), ICGC-LIRI-JP (HCCDB18).

#### Gene Correlation Analysis for apoptosis using UALCAN

The interactive web portal UALCAN (http://ualcan.path.uab.edu) [34] is a comprehensive web database for investigating complete genetic or molecular data of cancers and allows in-silico validation of genes of interest.

UALCAN was utilized to analyze the corresponding expression of the target gene involved in apoptosis (BAX, Bcl-2, CASP-3) in tumor and normal specimens based on The Cancer Genome Atlas (TCGA) database. The corresponding expression of the gene of interest can be investigated *per* the different tumor subgroups (e.g., tumor stage, histological subtype, and sex). Our data were analyzed as sample type: normal vs. primary liver HCC.

**Scheme 1:**
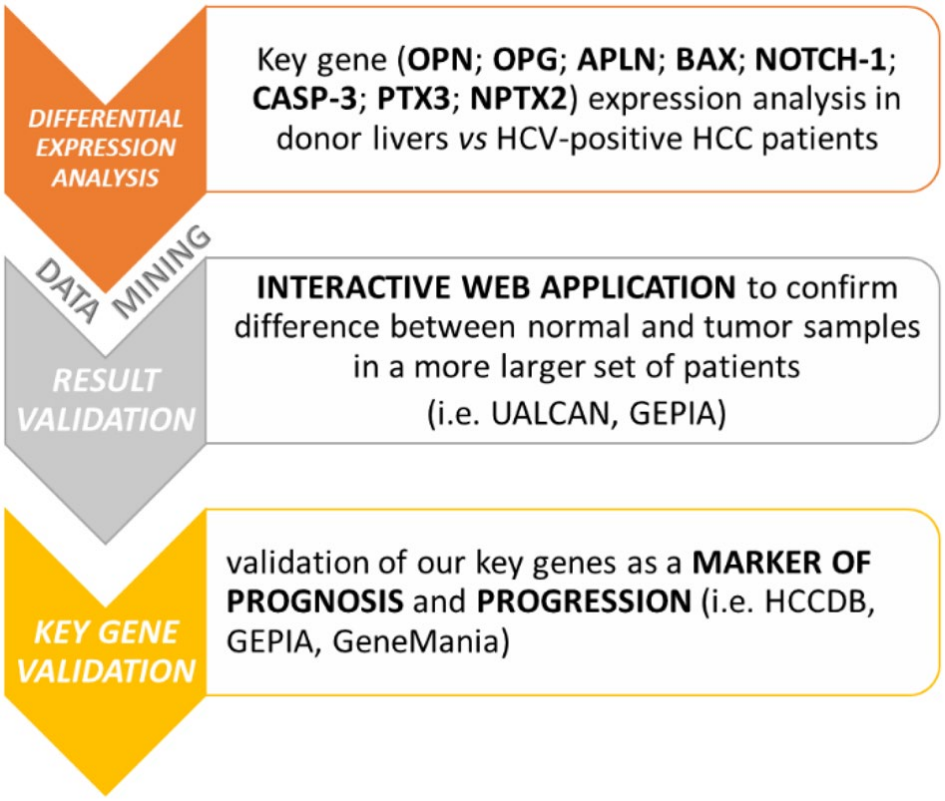
Step-by-step data analysis workflow designed for data mining approach

A scheme representing a detailed step-by-step workflow was reported (Scheme 1).

## Results

### Liver recipients and donor characteristics

The baseline demographic and clinical characteristics of the liver recipients, collected during the enrolment and including the patient’s sex, age, causes of liver cirrhosis, and liver disease severity score before transplant, as well as hepatic biochemical parameters and creatinine of LR and LD patients were illustrated in Table 2. Alkaline phosphatase (ALP, 128.3 ± 16.8 UI/L), bilirubin (1.3 ± 0.2 mg/dL), the international normalized ratio (INR, 1.2 ± 0.05%), fibrinogen (223.9 ± 15.9 mg/dL), and albumin (3.8 ± 0.2 g/dL) were available only for LR patients. Recipients were mainly males (80%) and the indication for liver transplantation was HCC.

**Table 2:** Clinical characteristic of HCV-related HCC patients and donor graft subjects

	Donor liver grafts (LD, n = 14)	Recipient liver (LR, n = 10)	p
Age, yrs	62.1 ± 17.3	59.4 ± 1.8	
Gender	–	8 M/2F	
*Diagnosis*			
HCC + Cirrosis +		8/10	
HCV HCC +	–	1/10	
Cirrosis + HBV		1/10	
HCC + HCV			
*Genotype*			
1a		2/10	
1b		3/10	
2c	–	1/10	
HCV-RNA-negative		2/10	
Not typed		2/10	
Total MELD score	0	8.6 ± 0.6	
AST, UI/L	10.1 ± 1.5	110.0 ± 19.9	< 0.0001
ALT, UI/L	7.2 ± 1.6	100.4 ± 20.6	< 0.0001
GGT, UI/L	34.4 ± 12.7	139.2 ± 23.3	0.0004
Creatinine	1.2 ± 0.2	0.85 ± 0.06	ns

*HCC* hepatocellular carcinoma, *HCV* Hepatitis C virus, *HBV* Hepatitis B virus, *MELD score* model for end-stage liver disease, *AST* aspartate aminotransferase, *ALT* alanine aminotransferase, *GGT* Gamma-glutamyl transferase

### Multi-mRNA biomarkers and HCC: step-wise analysis

**Fig. 1:**
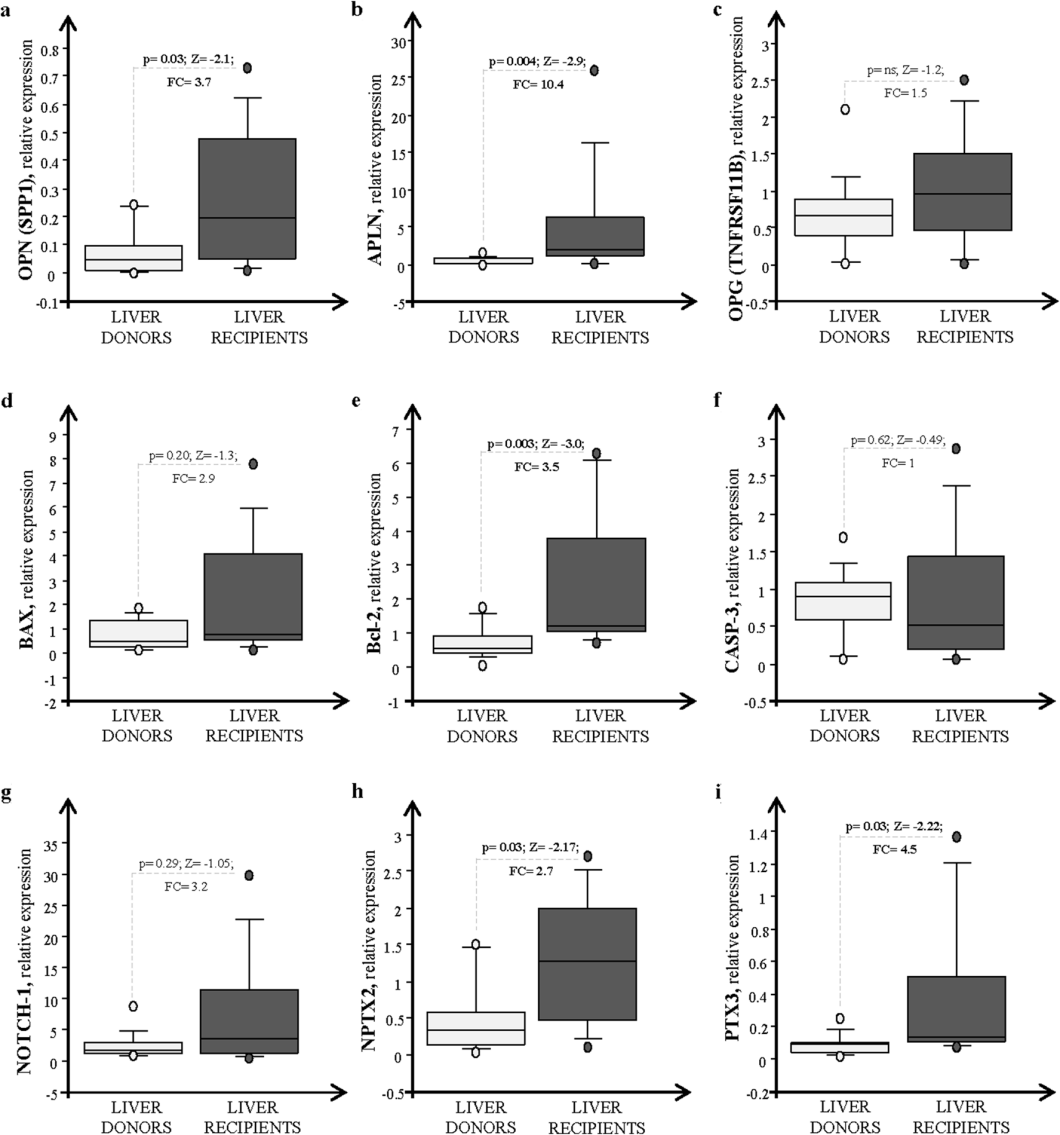
Transcriptional profiling of key genes **a** OPN/SPP1; **b** APLN; **c** OPG/TNFRSF11B; **d** BAX; **e** Bcl-2; **f** CASP-3; **g** NOTCH-1; **h** NPTX2 and **i** PTX3 in hepatic biopsies of healthy donors (light grey box-plots) and recipients (dark grey bar box-plots) patients. Each box consists of five horizontal lines displaying the 10th, 25th, 50th (median), 75th, and 90th percentiles of the variable. All values, above the 90th percentile and below the 10th percentile, are plotted separately

In Fig. 1 are depicted the transcriptional profiles of the key genes obtained by our previous works analyzed by Mann–Whitney U test [15–18]. As already mentioned above, the mRNA expression of OPN and Apelin resulted significantly higher in liver samples obtained from recipients than in those from donors. Among apoptotic markers, only Bcl-2 mRNA expression levels resulted significantly higher in LR with respect to LD, while BAX, CASP-3, and NOTCH-1 did not significantly change in both groups even though all genes were highly expressed in HCV-related HCC patients. On the contrary, the OPG expression didn’t result significantly different in LD and LR. Analyzing the PTX3 and NPTX2 transcripts, as independent markers of inflammation, their expression was significantly up-regulated in HCC tissues.

To evaluate the best predictive biomarkers in HCC between the key genes studied, in this work a multi mRNA-biomarker analysis was carried out.

Stepwise logistic model analysis was performed exploiting these variables and the best predictive biomarkers in our cohort of patients with HCC resulted in APLN (0.15(SE 0.05) p = 0.005) and OPN (0.96(SE 0.41) p = 0.003).

### Data mining result analysis

#### GeneMANIA analysis

**Fig. 2:**
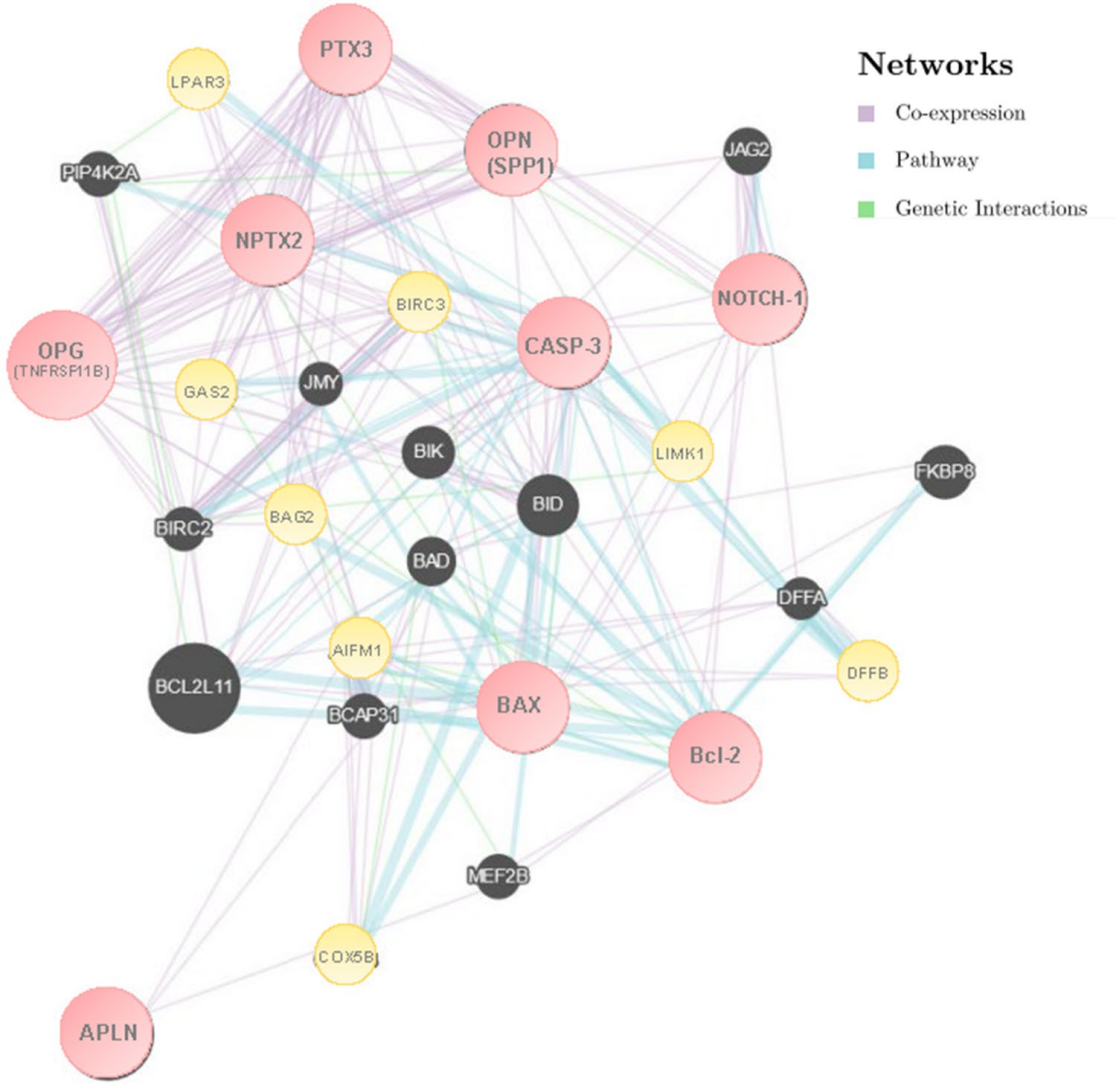
Interaction network of the key genes by GeneMANIA. The initial list of targeted genes and the type of connections between genes/proteins are illustrated in the network legend. In particular are depicted 9 nodes representing the key genes (light red dot); 20 critical interacting molecules (black dot) and 337 edges/links (purple: co-expression; cyan: pathway; green: genetic interactions). The 8 most representative critical interacting molecules were evidenced in light yellow. (Color figure online)

Protein–protein interaction network and functional annotation of genes were also evaluated by GeneMANIA. A visual analysis using this bioinformatic tool was performed to determine the potential mutual effects of the cancer-related targets studied. The Protein–protein interaction network consisted of 29 nodes, of which 20 critical interacting molecules and 337 edges/links, where nodes represent genes and edges/links represent networks (Fig. 2). In particular, among the 20 critical interacting molecules (black dot) the most representatives were evidenced in light yellow.

Physical/genetic interactions and co-expression are apparent among potentially affected genes mainly related to inflammatory and apoptotic processes.

#### GEPIA database

**Fig. 3:**
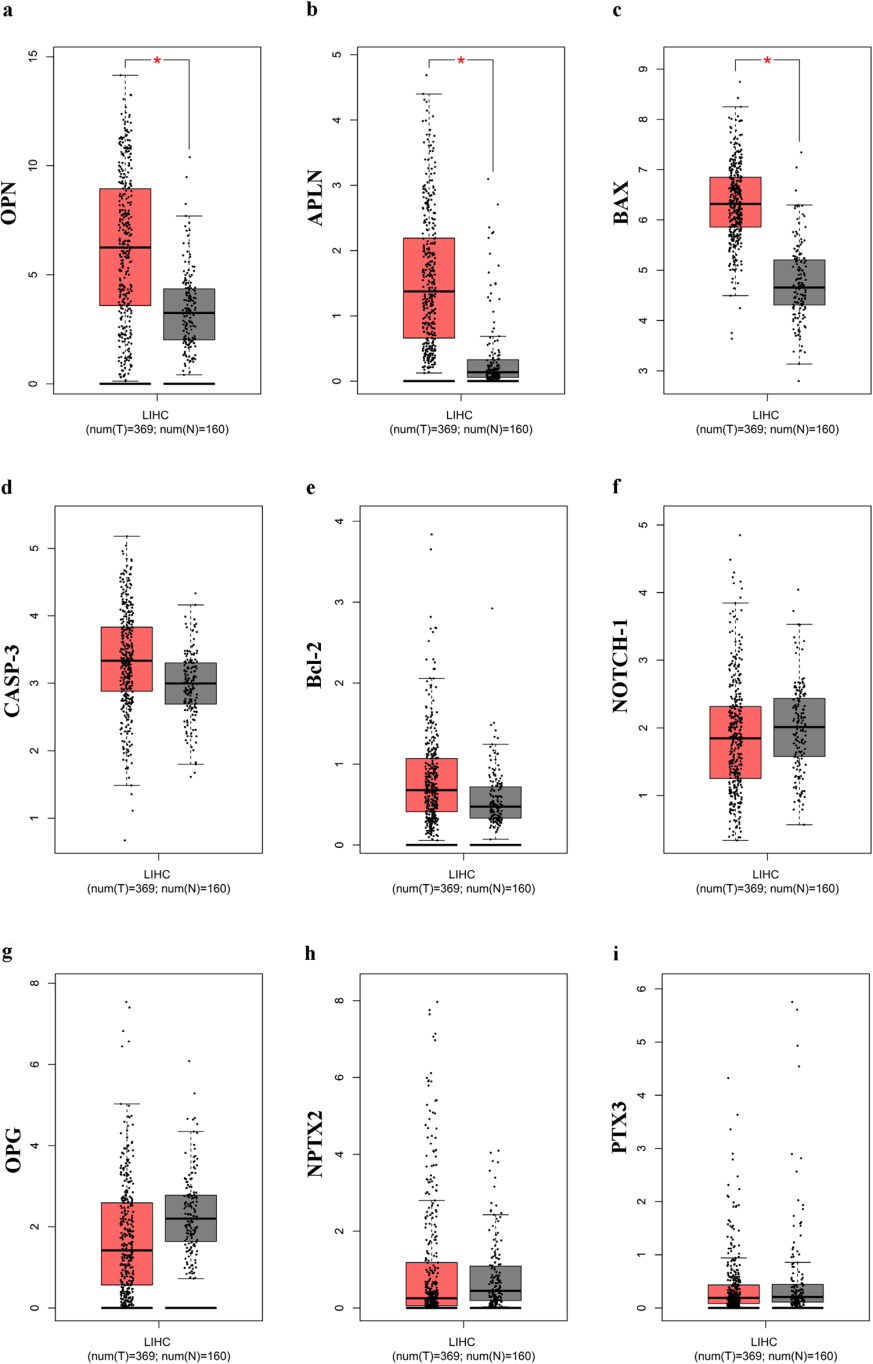
Box plot of key gene mRNA expression using GEPIA database in liver hepatocellular carcinoma (LIHC/HCC) patients: **a** OPN; **b** APLN; **c** BAX; **d** CASP-3; **e** Bcl-2; **f** NOTCH-1; **g** OPG; **h** NPTX2; **i** PTX3. Red boxplot: tumor tissues (n = 369); grey box plot: normal tissues (n = 160). (Color figure online)

Firstly, the GEPIA database was employed to identify mRNA expression of our key genes in HCC, namely Liver hepatocellular carcinoma (LIHC) in bioinformatics software, by a differential expression analysis (DIY Expression). For LIHC analysis GEPIA database use 369 tumor tissue samples and 160 normal tissue. Figure 3 depicted the expression levels of each key gene in the LIHC dataset, showing a significant up-regulation in HCC tumor samples compared with normal tissues for OPN (Fig. 3a), APLN (Fig. 3b), and BAX (Fig. 3c).

**Fig. 4:**
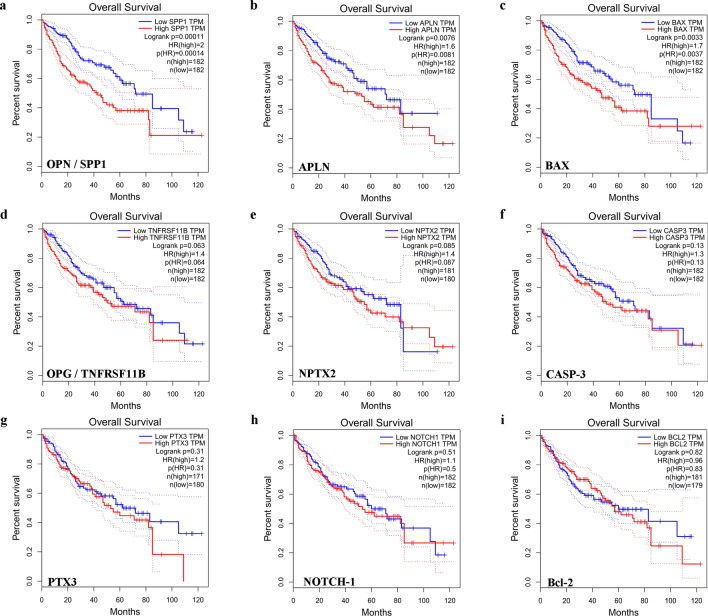
validation of hub genes prognostic values by GEPIA database. Overall survival (OS) curves for **a** OPN; **b** APLN; **c** BAX; **d** OPG; **e** NPTX2; **f** CASP-3; **g** PTX3; **h** NOTCH-1; **i** Bcl-2 in all cases of LIHC. Survival curves marked as complete lines, and 95% confidence interval of survival curves marked as dotted lines. Red represents high expression and blue represents low expression. (Color figure online)

The most important measures for survival plot in cancer studies include the overall survival (OS) curve, and the Disease-free survival (DSF or RFS, which correspond to the percentage of people in a study who have not died from a specific disease in a defined period). As reported in Fig. 4 only the 3 key genes [OPN (Fig. 4a), APLN (Fig. 4b), and BAX (Fig. 4c)] were significantly overexpressed and associated with worse OS of patients with LIHC cancer, confirming data obtained by the expression DIY analysis. Disease-free survival did not differ significantly (data not shown).

**Fig. 5:**
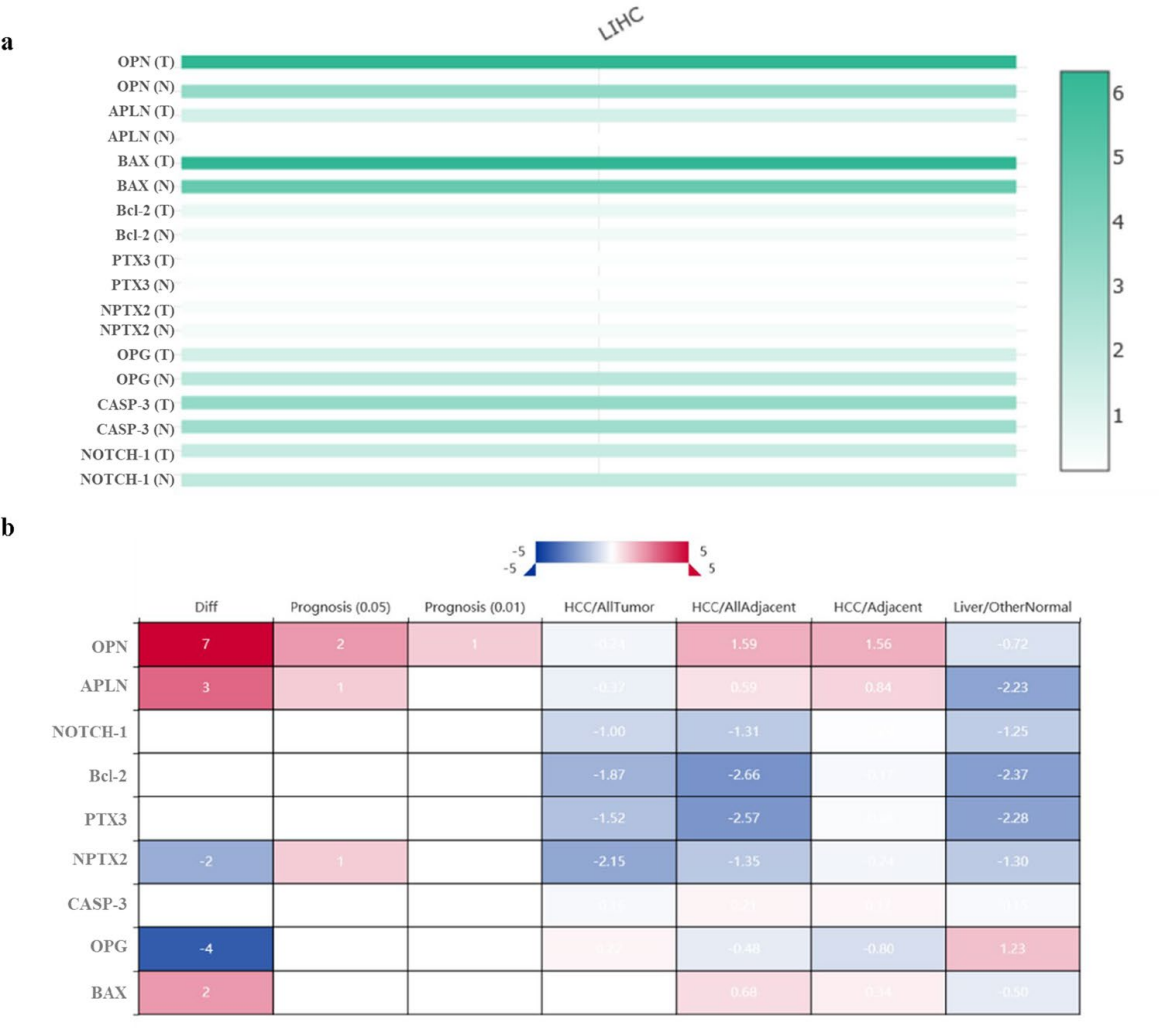
**a** Heat map showing the expression level of the key (OPN; APLN; BAX; OPG; NPTX2; CASP-3; PTX3; NOTCH-1; Bcl-2) in LIHC, and normal hepatic tissue based on TCGA normal and GTEx data analyzed by GEPIA web server. The T represents LIHC tumor tissues and the N represents normal liver tissue. Light green: low expression, dark green: higher expression rate. **b** Transcript levels of key genes in LIHC analyzed with HCCDB generating a multi-gene summary. Threshold setting: *p* value: 0.05; fold change: all; gene rank: top 10%. Red represents upregulation (unfavourable) and blue represents downregulation (favorable). The numbers in the coloured cells represent the numbers of dataset meeting the threshold. Each label indicates: *Diff:* the number of differentially expressed datasets; Prognosis: the number of significant datasets by survival analysis; *HCC/AllTumor*: Red/Blue for positive/negative fold change in log_2_ scale by comparing HCC with all tumors (TCGA data); *HCC/AllAdjacent*: Red/Blue for positive/negative fold change in log2 scale by comparing HCC with all adjacent samples (TCGA data); *HCC/Adjacent*: Red/Blue for positive/negative fold change in log_2_ scale by comparing HCC with adjacent samples (HCCDB data); *Liver/OtherNormal*: Red/Blue for positive/negative fold change in log_2_ scale by comparing liver with normal tissues (GTEx&TCGA data). (Color figure online)

Then, we used the “Multiple Gene Comparison” function of GEPIA to analyze the expression of the key genes in LIHC and normal hepatic tissues by the TCGA database and the results showed that OPN and BAX were about 6 times higher in hepatic tumor tissue than that in normal (Fig. 5a).

#### HCCDB database analysis

The consistently differentially expressed gene in the LIHC dataset was the OPN, as expected, which resulted the most up-regulated gene in 7 of the datasets analyzed by HCCDB (Fig. 5b). None of them was classified as prognostic genes. A new figure (Panel A, B, C) representing the expression levels of our key genes across the dataset used was also reported in Supplementary materials (Suppl. 1).

#### UALCAN interactive web analysis

**Fig. 6:**
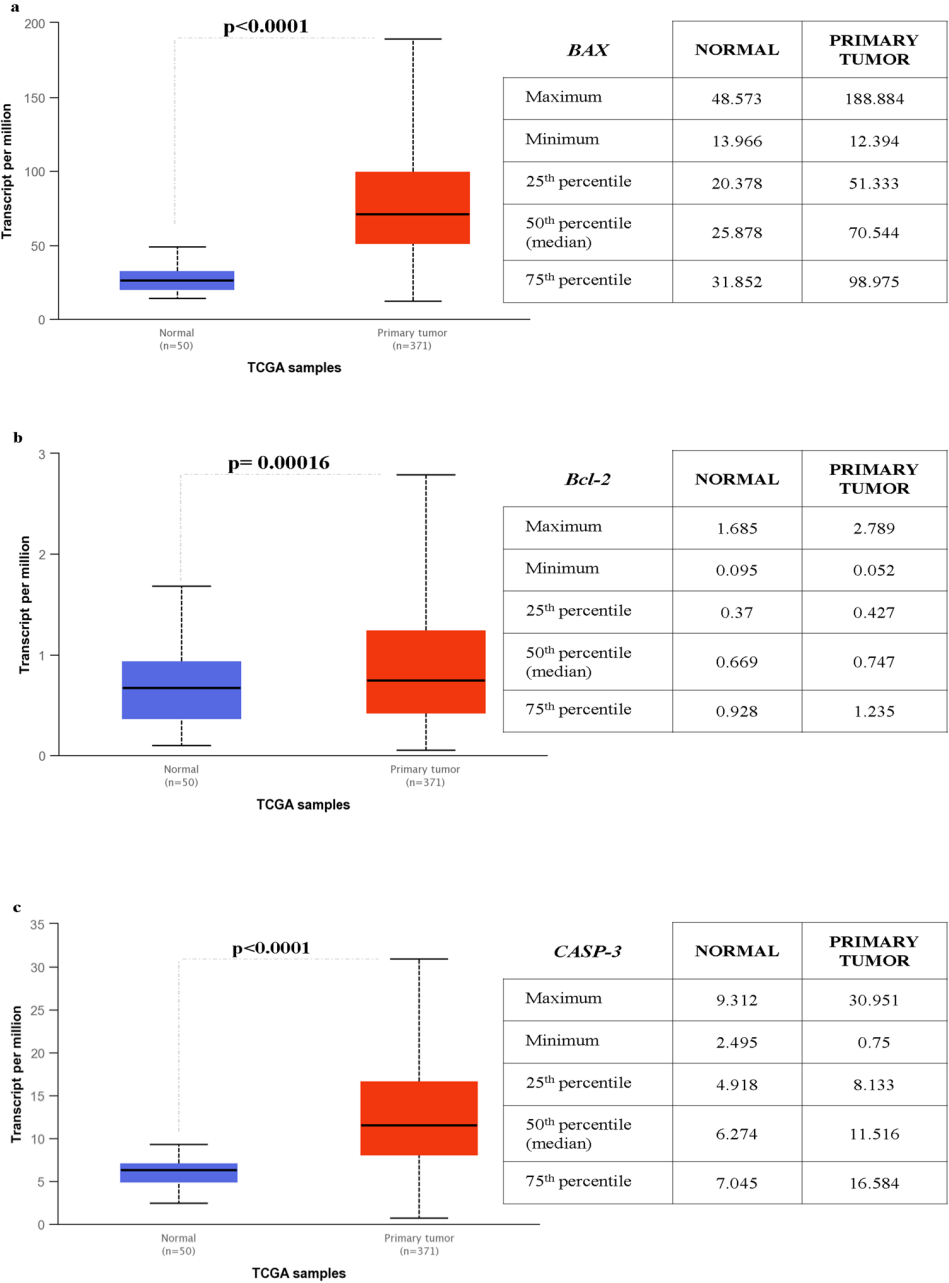
expression of key genes based on sample type and apoptotic process performed with the UALCAN database in LIHC (Liver hepatocellular carcinoma). **a** BAX; **b** Bcl-2; **c** CASP-3 Red boxplot: primary tumor tissues (n = 371); blue boxplot: normal tissues (n = 50). Besides a table reporting the expression value related to median, upper/lower quartile and maximum/minimum (TPM) of each gene analysed. (Color figure online)

To analyze the key genes mRNA expression in the apoptosis process, we use the UALCAN website. As reported in Fig. 6 the mRNA expression levels of apoptotic markers Bcl-2, BAX and CASP-3 in normal and primary tumor liver tissues were explored. Compared with those in normal tissues, the expression levels of both of them were significantly higher (Fig. 6), and these results were consistent with those observed in the previous analysis, corroborating their role in apoptosis during HCC development (Suppl. 2).

## Discussion

HCC is the main pathological type of liver cancer having high morbidity and mortality worldwide. To date, surgical treatments remain the major interventional measures that can effectively improve the prognosis of early HCC patients; however, a large number of HCC patients are diagnosed at an advanced stage making them unsuitable for treatments and exposing them to recurrence and metastasis.

Thanks to the development of high-throughput technologies, several molecular data have been used to delineate the alterations in HCC at multiple levels [35–38], but additional biomarkers for the early detection of HCC are also required and this is the first study that carried out a comprehensive strategy of data mining in this pathology.

In fact, given the significant role deriving from our previous studies [19–22] for each biomarker analyzed (APLN, OPN, NOTCH-1, CASP-3, Bcl-2, BAX, PTX3, and NPTX2), we believed important to evaluate which of these key genes could have a predominant role in the HCC pathology, exploiting statistical analysis and in-silico tools to build a multi-biomarker model. Firstly, we performed a stepwise regression analysis (logistic model) using our mRNA-datasets which revealed that higher expression levels of OPN and APLN were positively associated with the HCC. We are pleased to note that our results were confirmed with GEPIA, GeneMANIA, and HCCDB tools that gathered together public online databases derived from independent studies [39, 40]. The mRNA expression analysis from all these databases demonstrated that these two markers were conspicuously upregulated in HCC tissues with their corresponding normal tissues.

Osteopontin, a glycoprotein of the extracellular matrix, and apelin, an endogenous peptide expressed in numerous tissues able to regulate physiological and pathological processes, turn out to have a potential role in liver cancer development and progression [41]. The present study is the first multi-biomarker study that analyzes these set of key genes in HCC confirming the hypothesis that OPN and APLN might play a relevant role in the mechanisms that drive oncogenesis and in the early diagnosis of HCC [18, 21, 42–44].

Moreover, the physical interactions and pathways analyzed with GeneMANIA, depicted that apoptosis and inflammation markers (i.e. BAX, Bcl-2, CASP-3, PTX3) shares consolidated pathways as the regulation of intrinsic/extrinsic apoptotic signalling and signal transduction [44–48]. All the interaction networks shared among our selected biomarkers with other genes could have a promising role in cancer pathology and may act as a potential biomarker of progression and/or diagnosis/prognosis [49–56]. These data about the apoptotic process were also confirmed by UALCAN, a comprehensive web database for investigating complete genetic/molecular data of cancers, that as expected showed BAX, CASP-3, and Bcl-2 genes as important regulators of apoptosis and unfavorable prognosis.

The prognostic relevance of OPN and APLN in HCC was further investigated by analyzing with GEPIA database, the survival plot (overall survival), and the disease-free survival that reported that elevated OPN and APLN gene expression were associated with a poor survival rate. On the contrary, their lower expression levels were correlated with more favorable conditions. Moreover, given the high heterogeneity nature of HCC, performing bioinformatic analysis of the same genes with the HCCDB database we detected that only OPN is the most consistently differentially expressed gene across multiple HCC expression datasets. Integrating data from GTEx and TCGA, we provide a global differential landscape able to identify consistent patterns that would be beneficial for identifying reliable biomarkers as OPN.

## Conclusions

The present study was conducted to explore the expression profile and the role of the key genes in HCC using onco-informatics analysis. Extensive data mining from publicly available databases revealed that APLN and mainly OPN were upregulated in HCC with respect to their respective normal tissues. Thus, we evidenced that OPN might have a potential function as an important tumor marker-driven oncogenesis being associated with poor prognosis of HCC patients. Although the results presented in this paper were based on rigorous processes and appropriate methodology, the study inevitably has several limitations. First, the main is the recruitment of patients meeting the inclusion criteria that were limited to a single Center and a larger number of patients is required to confirm the present findings; second, the number of key genes analyzed could be enlarged. Finally, a common limitation of in-silico investigations consists of using a database with limited clinical data concerning patient outcomes. Anyway, further bioinformatics analysis methods to implement basic research in this field are needed to explore and better understand the exact molecular mechanism and the specific pathways that mediate its role in the development of HCC.

## Supplementary Information

Below is the link to the electronic supplementary material.

Supplementary file1 **Supplementary Material Fig.1** Expression pattern of key genes in hepatocellular carcinoma (HCC) and normal tissues in the HCCDB database. The expression pattern view displayed the patterns in the archived HCC datasets, tissues in GTEx and tumors in TCGA. On the left: clinical cohorts and identification of HCCDB database used. On the right. Boxplot representation. In red: HCC samples; in blue: adjacent samples; in cyan: cirrhotic samples; in orange: healthy samples (PDF 453 kb)

Supplementary file2 **Fig. 2** involvement of BAX, Bcl-2, CASP-3 and PTX3 genes during the apoptotic process with UALCAN data-web. Table to explore TGCA database expression data and survival information for apoptotic pathway and related genes: **a**) Bcl-2, **b**) BAX **c**) CASP-3 and **d**) PTX3 (PNG 1994 kb)

## Data Availability

The data that support the findings of this study are available in IFC-CNR. Anyway, all data generated or analyzed during this study are included in this published article.
