# Supplementary material for: Data mining of key genes expression in hepatocellular carcinoma: novel potential biomarkers of diagnosis prognosis or progression
Source: Clin Exp Metastasis. 2022 Apr 16;39(4):589–602. doi: 10.1007/s10585-022-10164-9 (PMC9338913; doi:10.1007/s10585-022-10164-9)

OPN

Dataset	P-value	Type	Nums	Mean	STD	IQR
HCCDB1	0.0008682	HCC	100	10.80	1.959	2.957
		Adjacent	97	10.03	1.125	1.395
HCCDB3	1.220e-15	HCC	268	6.536	6.529	10.32
		Adjacent	243	2.917	2.575	2.683
		Cirrhotic	40	10.62	5.418	8.601
		Healthy	6	1.485	0.9042	0.9250
HCCDB4	2.550e-21	HCC	240	9.125	2.094	3.535
		Adjacent	193	7.657	0.6486	0.6970
HCCDB6	6.780e-24	HCC	225	8.483	2.481	3.910
		Adjacent	220	6.430	1.352	1.666
HCCDB7	0.0002958	HCC	80	12.38	1.564	2.296
		Adjacent	82	11.61	1.019	1.449
HCCDB11	0.006040	HCC	88	9.275	2.311	3.610
		Adjacent	48	8.389	1.390	2.101
HCCDB12	0.002227	HCC	81	11.49	2.947	4.429
		Adjacent	80	10.29	1.800	2.235
HCCDB13	1.550e-9	HCC	228	9.709	2.949	4.725
		Adjacent	168	8.279	1.591	2.264
HCCDB15	1.260e-10	HCC	351	11.20	3.620	5.285
		Adjacent	49	9.362	1.302	1.620
HCCDB16	0.00002350	HCC	60	10.87	2.004	3.091
		Adjacent	60	9.438	1.521	2.216
HCCDB17	0.2406	HCC	115	9.195	3.713	3.768
		Adjacent	52	8.521	3.273	3.149
HCCDB18	6.570e-22	HCC	212	6.260	3.170	5.073
		Adjacent	177	3.720	1.475	1.810

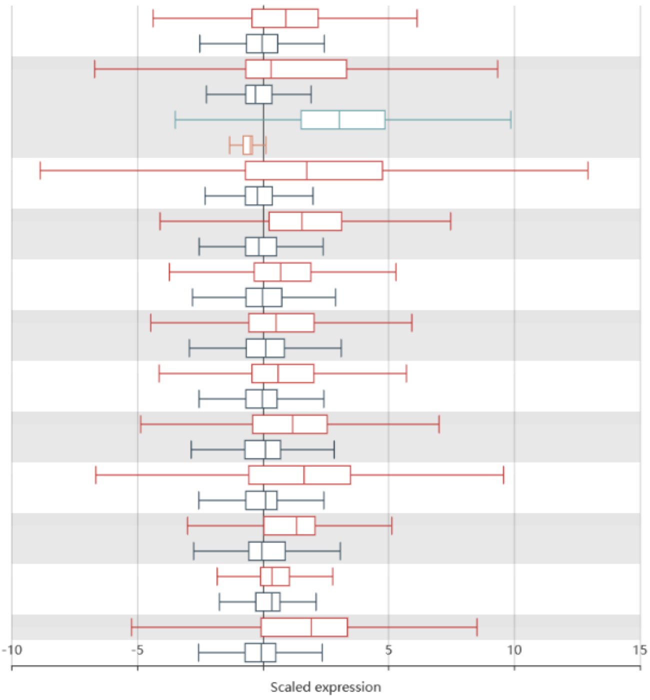

OPG

Dataset	P-value	Type	Nums	Mean	STD	IQR
HCCDB1	2.300e-18	HCC	100	7.116	1.389	1.824
		Adjacent	97	8.647	0.5528	0.6820
HCCDB4	0.9318	HCC	240	6.462	0.8095	0.8865
		Adjacent	193	6.467	0.3687	0.4934
HCCDB6	0.03971	HCC	225	4.059	0.9332	1.025
		Adjacent	220	4.208	0.5327	0.6505
HCCDB7	0.07676	HCC	80	10.46	0.7926	1.109
		Adjacent	82	10.67	0.6387	0.7940
HCCDB11	0.09086	HCC	88	7.305	1.679	3.184
		Adjacent	48	7.848	1.821	2.700
HCCDB12	0.0004615	HCC	81	8.141	1.532	1.779
		Adjacent	80	8.877	1.027	0.8740
HCCDB13	1.240e-34	HCC	228	6.344	1.599	2.751
		Adjacent	168	8.021	0.7877	1.035
HCCDB15	8.450e-33	HCC	351	6.234	2.409	3.290
		Adjacent	49	8.587	0.7438	0.8400
HCCDB16	0.005343	HCC	60	6.693	0.8095	1.103
		Adjacent	60	7.042	0.4919	0.5173
HCCDB17	0.01057	HCC	115	7.039	0.6529	0.6100
		Adjacent	52	7.354	0.7526	0.9025
HCCDB18	2.190e-18	HCC	212	1.679	1.253	1.910
		Adjacent	177	2.625	0.7351	0.8700

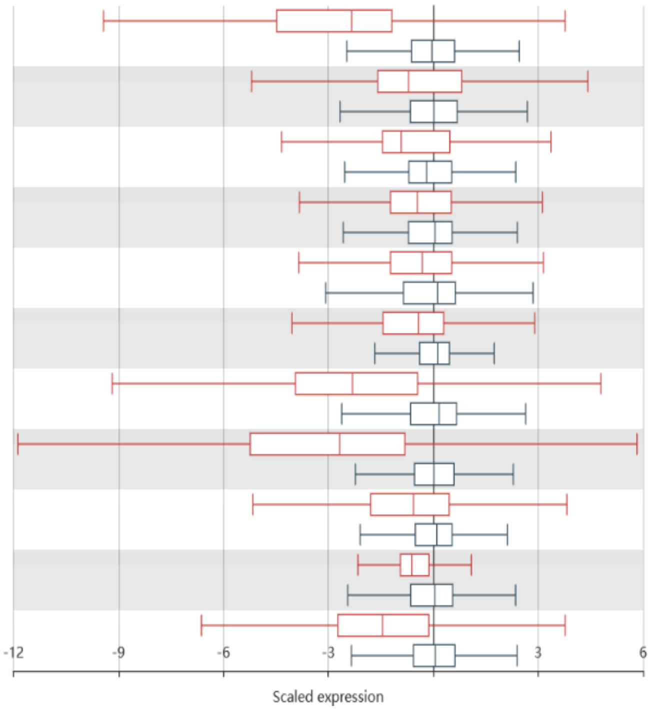

APLN

Dataset	P-value	Type	Nums	Mean	STD	IQR
HCCDB1	1.550e-25	HCC	100	8.172	1.075	1.396
		Adjacent	97	6.619	0.5905	0.8198
HCCDB3	4.740e-24	HCC	268	0.8995	0.7737	0.7079
		Adjacent	243	0.3601	0.1928	0.1875
		Cirrhotic	40	0.4215	0.1648	0.2170
		Healthy	6	0.1953	0.05818	0.07425
HCCDB4	6.500e-30	HCC	240	6.607	0.3331	0.3281
		Adjacent	193	6.303	0.1579	0.2160
HCCDB11	0.6169	HCC	88	7.177	1.536	2.329
		Adjacent	48	7.317	1.566	2.800
HCCDB12	0.001400	HCC	81	8.045	0.9870	1.356
		Adjacent	80	7.575	0.8421	1.004
HCCDB13	3.580e-21	HCC	228	4.802	0.4686	0.5100
		Adjacent	168	4.452	0.1961	0.2242
HCCDB15	1.450e-29	HCC	351	7.154	1.587	2.385
		Adjacent	49	3.512	1.223	1.820
HCCDB16	0.000005300	HCC	60	6.882	0.3794	0.4703
		Adjacent	60	6.579	0.3117	0.3639
HCCDB17	0.000002310	HCC	115	7.286	0.5926	0.6750
		Adjacent	52	6.827	0.5304	0.4425
HCCDB18	6.930e-39	HCC	212	1.113	0.7846	0.8725
		Adjacent	177	0.2230	0.2776	0.1600

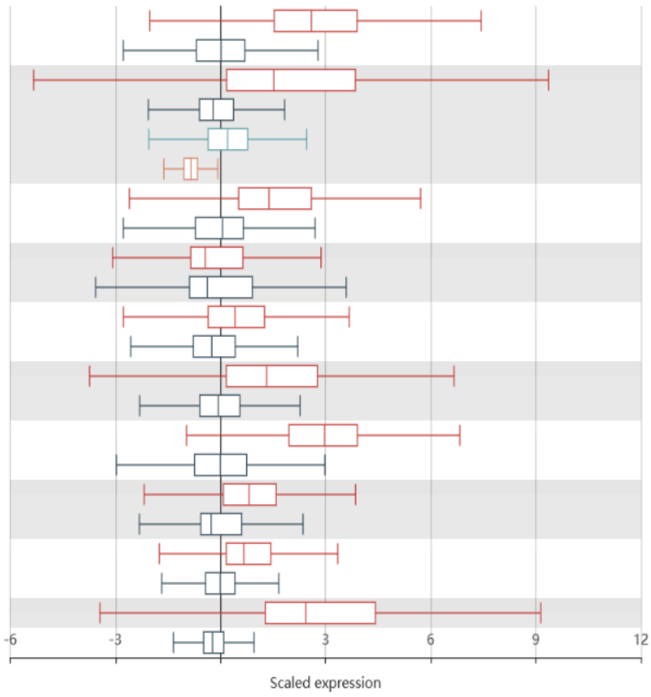

Bcl2

Dataset	P-value	Type	Nums	Mean	STD	IQR
HCCDB1	3.630e-7	HCC	100	6.969	0.7709	1.139
		Adjacent	97	7.451	0.4763	0.6069
HCCDB3	4.830e-13	HCC	268	0.3642	0.2299	0.2943
		Adjacent	243	0.5153	0.2296	0.2510
		Cirrhotic	40	0.7936	0.2960	0.4184
		Healthy	6	0.2746	0.1245	0.1821
HCCDB4	0.0001365	HCC	240	6.747	0.1654	0.1942
		Adjacent	193	6.691	0.1354	0.1723
HCCDB6	0.000002010	HCC	225	3.736	0.2100	0.2370
		Adjacent	220	3.837	0.2303	0.2229
HCCDB7	0.008998	HCC	80	12.71	0.5937	0.7652
		Adjacent	82	12.94	0.5113	0.7088
HCCDB11	0.00001790	HCC	88	6.732	0.5583	0.7929
		Adjacent	48	7.198	0.5812	0.6789
HCCDB12	0.01709	HCC	81	9.188	0.6421	0.9625
		Adjacent	80	9.436	0.6597	0.8945
HCCDB13	0.00001800	HCC	228	4.277	0.1986	0.2311
		Adjacent	168	4.361	0.1844	0.2078
HCCDB15	0.009478	HCC	351	6.783	1.104	1.395
		Adjacent	49	7.118	0.7807	1.000
HCCDB16	0.7184	HCC	60	7.113	0.3699	0.4210
		Adjacent	60	7.137	0.3481	0.4738
HCCDB17	0.2807	HCC	115	7.144	0.1803	0.2250
		Adjacent	52	7.114	0.1653	0.2088
HCCDB18	0.9916	HCC	212	0.9394	0.4995	0.6525
		Adjacent	177	0.9389	0.4633	0.5900

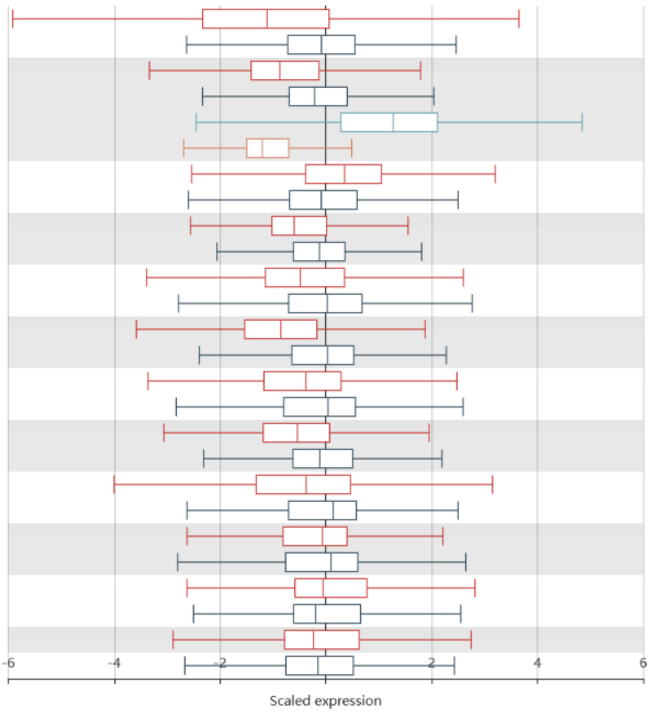

CASP-3

Dataset	P-value	Type	Nums	Mean	STD	IQR
HCCDB1	1.120e-8	HCC	100	9.449	0.4131	0.5672
		Adjacent	97	9.161	0.2346	0.3244
HCCDB3	1.010e-15	HCC	268	1.244	0.4225	0.5150
		Adjacent	243	1.005	0.1920	0.2235
		Cirrhotic	40	1.158	0.1959	0.2920
		Healthy	6	0.9072	0.2019	0.2980
HCCDB4	3.610e-7	HCC	240	7.906	0.3137	0.4195
		Adjacent	193	7.780	0.1845	0.2602
HCCDB6	1.140e-20	HCC	225	5.647	0.5670	0.7770
		Adjacent	220	5.217	0.3133	0.3420
HCCDB7	0.00005660	HCC	80	12.65	0.4187	0.6277
		Adjacent	82	12.30	0.6254	0.7983
HCCDB11	0.2127	HCC	88	6.975	0.9148	1.399
		Adjacent	48	7.177	0.8917	1.287
HCCDB12	0.5117	HCC	81	11.64	0.4091	0.5450
		Adjacent	80	11.60	0.2815	0.3445
HCCDB13	0.002537	HCC	228	5.543	0.6067	0.8598
		Adjacent	168	5.391	0.3899	0.4695
HCCDB15	4.920e-8	HCC	351	9.306	0.6376	0.7700
		Adjacent	49	8.983	0.3059	0.3500
HCCDB16	0.5821	HCC	60	7.450	0.2493	0.3204
		Adjacent	60	7.471	0.1577	0.1671
HCCDB17	0.0002576	HCC	115	8.896	0.3309	0.3825
		Adjacent	52	9.054	0.2086	0.2937
HCCDB18	1.550e-20	HCC	212	3.564	0.5414	0.6900
		Adjacent	177	3.117	0.3445	0.4400

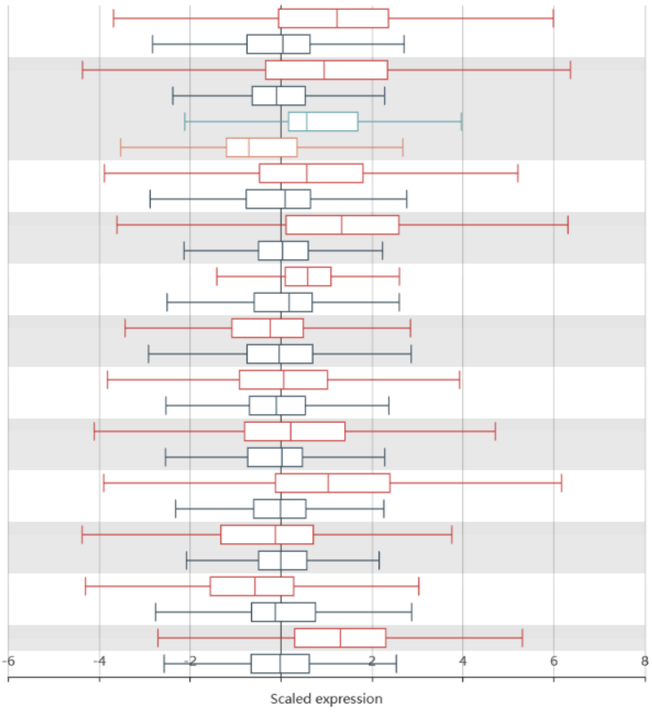

BAX

Dataset	P-value	Type	Nums	Mean	STD	IQR
HCCDB1	8.520e-10	HCC	100	10.15	0.5507	0.7223
		Adjacent	97	9.732	0.3384	0.3962
HCCDB3	2.470e-15	HCC	268	2.179	0.9177	1.035
		Adjacent	243	1.642	0.5255	0.5470
		Cirrhotic	40	2.076	0.7563	1.041
		Healthy	6	0.8903	0.3389	0.5185
HCCDB4	1.010e-9	HCC	240	7.075	0.2644	0.2833
		Adjacent	193	6.938	0.1904	0.1871
HCCDB6	2.340e-14	HCC	225	4.331	0.5666	0.6955
		Adjacent	220	3.988	0.3044	0.3604
HCCDB7	0.009048	HCC	80	11.71	1.014	1.548
		Adjacent	82	11.29	1.033	1.602
HCCDB11	0.6343	HCC	88	7.143	0.8087	0.9388
		Adjacent	48	7.055	1.126	1.740
HCCDB12	0.6913	HCC	81	10.79	0.7449	0.8690
		Adjacent	80	10.84	0.7718	0.7862
HCCDB13	1.340e-8	HCC	228	6.523	0.5216	0.6747
		Adjacent	168	6.250	0.4129	0.4943
HCCDB15	1.360e-14	HCC	351	10.10	0.7355	0.9600
		Adjacent	49	9.292	0.5290	0.6100
HCCDB16	0.00001120	HCC	60	8.846	0.2963	0.4911
		Adjacent	60	8.631	0.2072	0.2521
HCCDB17	0.9096	HCC	115	7.197	0.1639	0.2100
		Adjacent	52	7.201	0.1911	0.2200
HCCDB18	4.500e-34	HCC	212	4.967	0.6702	0.9850
		Adjacent	177	4.111	0.5846	0.7200

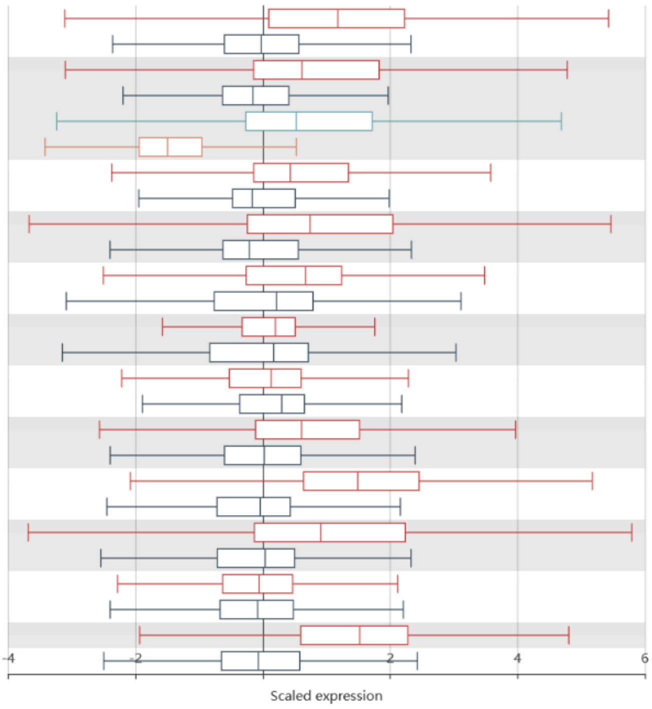

NOTCH-1

Dataset	P-value	Type	Nums	Mean	STD	IQR
HCCDB1	0.00007140	HCC	100	6.731	0.2672	0.3508
		Adjacent	97	6.861	0.1692	0.2178
HCCDB3	0.2004	HCC	268	0.3733	0.1568	0.1650
		Adjacent	243	0.3877	0.09122	0.1000
		Cirrhotic	40	0.3737	0.07493	0.1062
		Healthy	6	0.5084	0.09264	0.1418
HCCDB4	7.810e-12	HCC	240	7.921	0.5642	0.7574
		Adjacent	193	7.620	0.3040	0.4363
HCCDB6	0.0006642	HCC	225	5.094	0.5542	0.6640
		Adjacent	220	5.241	0.3204	0.4188
HCCDB11	0.3828	HCC	88	6.997	0.8155	1.064
		Adjacent	48	7.135	0.9095	1.431
HCCDB12	0.4644	HCC	81	6.143	0.9855	0.9850
		Adjacent	80	6.242	0.7188	0.8482
HCCDB13	0.7714	HCC	228	4.026	0.1916	0.2125
		Adjacent	168	4.032	0.1935	0.2525
HCCDB15	0.7301	HCC	351	9.314	0.9513	1.215
		Adjacent	49	9.346	0.5297	0.7900
HCCDB16	0.05142	HCC	60	7.806	0.3505	0.4473
		Adjacent	60	7.930	0.3378	0.2438
HCCDB17	0.001945	HCC	115	8.774	0.4754	0.5650
		Adjacent	52	8.992	0.3815	0.4325
HCCDB18	0.01398	HCC	212	1.927	0.6050	0.8700
		Adjacent	177	1.796	0.4350	0.4800

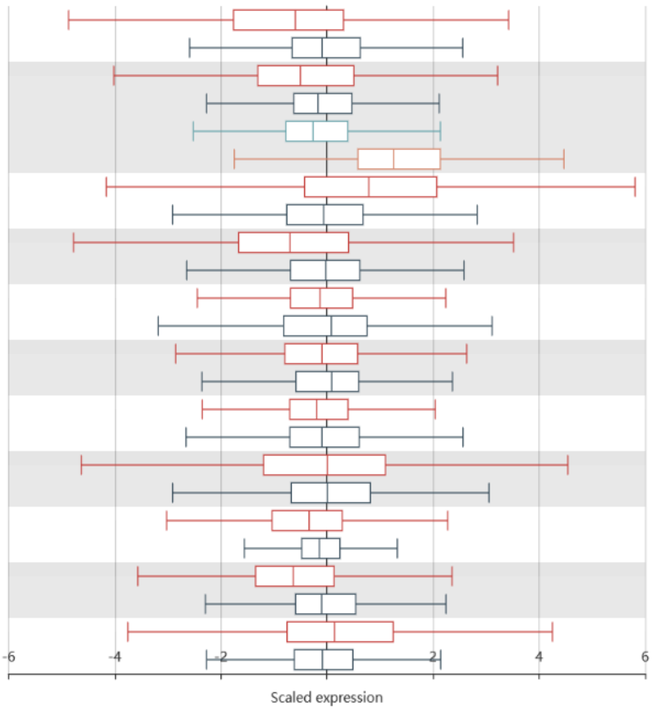

PTX3

Dataset	P-value	Type	Nums	Mean	STD	IQR
HCCDB1	0.5141	HCC	100	5.634	1.149	1.321
		Adjacent	97	5.759	1.498	2.258
HCCDB3	0.6245	HCC	268	0.2382	0.6827	0.1545
		Adjacent	243	0.2626	0.4233	0.2120
		Cirrhotic	40	0.9750	0.8339	0.6915
		Healthy	6	0.3017	0.3215	0.4760
HCCDB4	0.09513	HCC	240	5.661	0.1131	0.1480
		Adjacent	193	5.642	0.1194	0.1461
HCCDB6	0.01042	HCC	225	3.443	0.3807	0.2950
		Adjacent	220	3.365	0.2452	0.2308
HCCDB7	0.6365	HCC	80	10.60	0.8128	1.110
		Adjacent	82	10.66	0.8424	1.270
HCCDB11	0.01347	HCC	88	6.274	1.075	1.599
		Adjacent	48	6.864	1.410	2.796
HCCDB12	0.4656	HCC	81	5.341	1.332	1.628
		Adjacent	80	5.203	1.047	1.488
HCCDB13	0.00005430	HCC	228	4.134	0.8918	0.4690
		Adjacent	168	4.677	1.532	0.8505
HCCDB15	0.8283	HCC	351	3.192	1.733	2.285
		Adjacent	49	3.144	1.405	1.520
HCCDB16	0.1155	HCC	60	4.280	0.2355	0.2853
		Adjacent	60	4.217	0.1967	0.1775
HCCDB17	0.8333	HCC	115	6.414	0.1138	0.1450
		Adjacent	52	6.409	0.1268	0.1550
HCCDB18	0.2622	HCC	212	0.3985	0.5663	0.3300
		Adjacent	177	0.4765	0.7654	0.3500

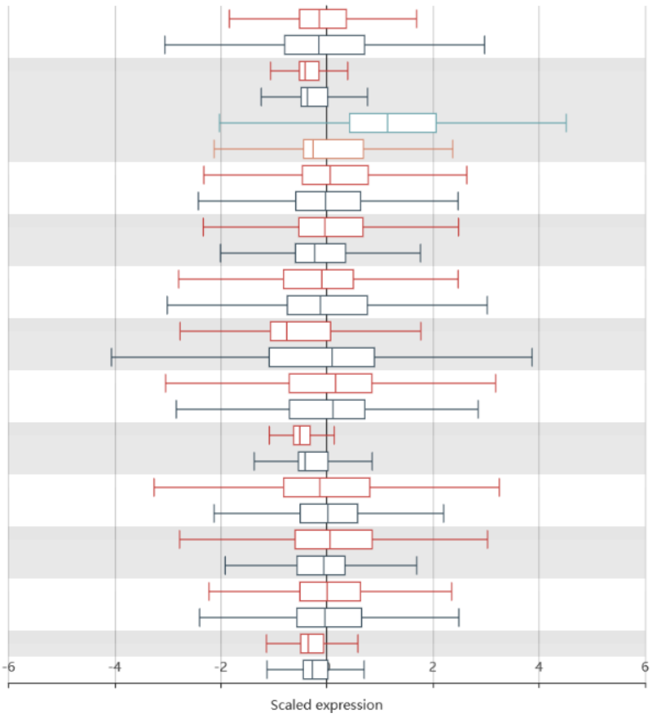

NPTX2

Dataset	P-value	Type	Nums	Mean	STD	IQR
HCCDB1	0.00002960	HCC	100	7.967	2.596	3.488
		Adjacent	97	9.174	0.9991	1.298
HCCDB3	0.03933	HCC	268	2.374	5.046	1.242
		Adjacent	243	1.711	1.355	1.303
		Cirrhotic	40	1.750	0.8240	0.6770
		Healthy	6	2.149	0.9856	0.2813
HCCDB4	9.010e-7	HCC	240	7.119	1.178	0.4610
		Adjacent	193	6.730	0.1973	0.2427
HCCDB6	0.000002450	HCC	225	4.563	1.591	0.8290
		Adjacent	220	4.037	0.3705	0.3990
HCCDB7	0.6474	HCC	80	11.49	0.8730	1.233
		Adjacent	82	11.55	0.7366	0.9350
HCCDB11	0.3307	HCC	88	7.145	1.493	2.628
		Adjacent	48	7.433	1.723	2.654
HCCDB12	0.00001700	HCC	81	4.377	2.101	3.290
		Adjacent	80	5.620	1.364	1.298
HCCDB13	0.2517	HCC	228	4.036	0.7962	0.2597
		Adjacent	168	4.104	0.3347	0.3888
HCCDB15	0.0006193	HCC	351	4.454	3.301	4.825
		Adjacent	49	5.405	1.442	2.050
HCCDB16	0.3455	HCC	60	6.351	0.3097	0.2835
		Adjacent	60	6.400	0.2492	0.2651
HCCDB17	0.1693	HCC	115	7.192	1.468	1.060
		Adjacent	52	7.397	0.4087	0.5300
HCCDB18	0.0005741	HCC	212	0.5079	1.300	0.2425
		Adjacent	177	0.8460	0.5069	0.5400

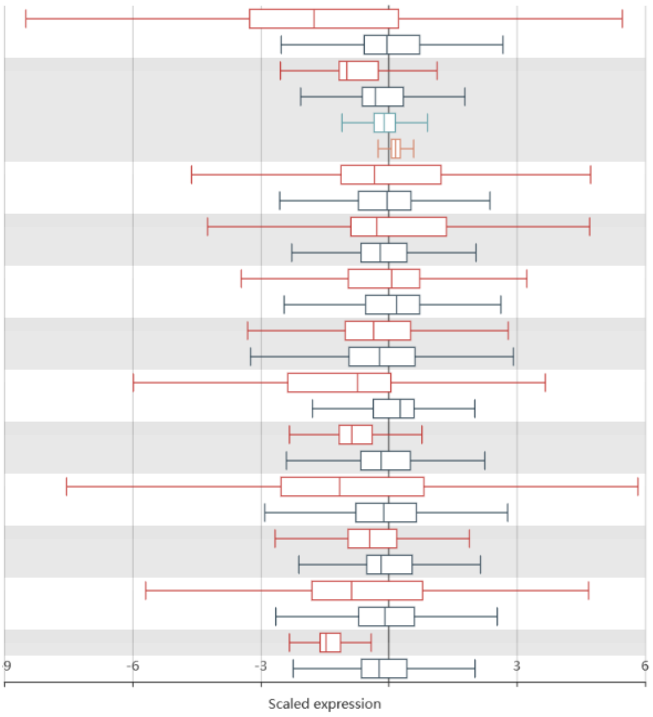

Supplement: Supplementary file 1 — Supplementary file1 Supplementary Material Fig.1 Expression pattern of key genes in hepatocellular carcinoma (HCC) and normal tissues in the HCCDB database. The expression pattern view displayed the patterns in the archived HCC datasets, tissues in GTEx and tumors in TCGA. On the left: clinical cohorts and identification of HCCDB database used. On the right. Boxplot representation. In red: HCC samples; in blue: adjacent samples; in cyan: cirrhotic samples; in orange: healthy samples (PDF 453 kb) [file 10585_2022_10164_MOESM1_ESM.pdf]
