# Supplementary figures and images for: Data mining of key genes expression in hepatocellular carcinoma: novel potential biomarkers of diagnosis prognosis or progression
Source: Clin Exp Metastasis. 2022 Apr 16;39(4):589–602. doi: 10.1007/s10585-022-10164-9 (PMC9338913; doi:10.1007/s10585-022-10164-9)

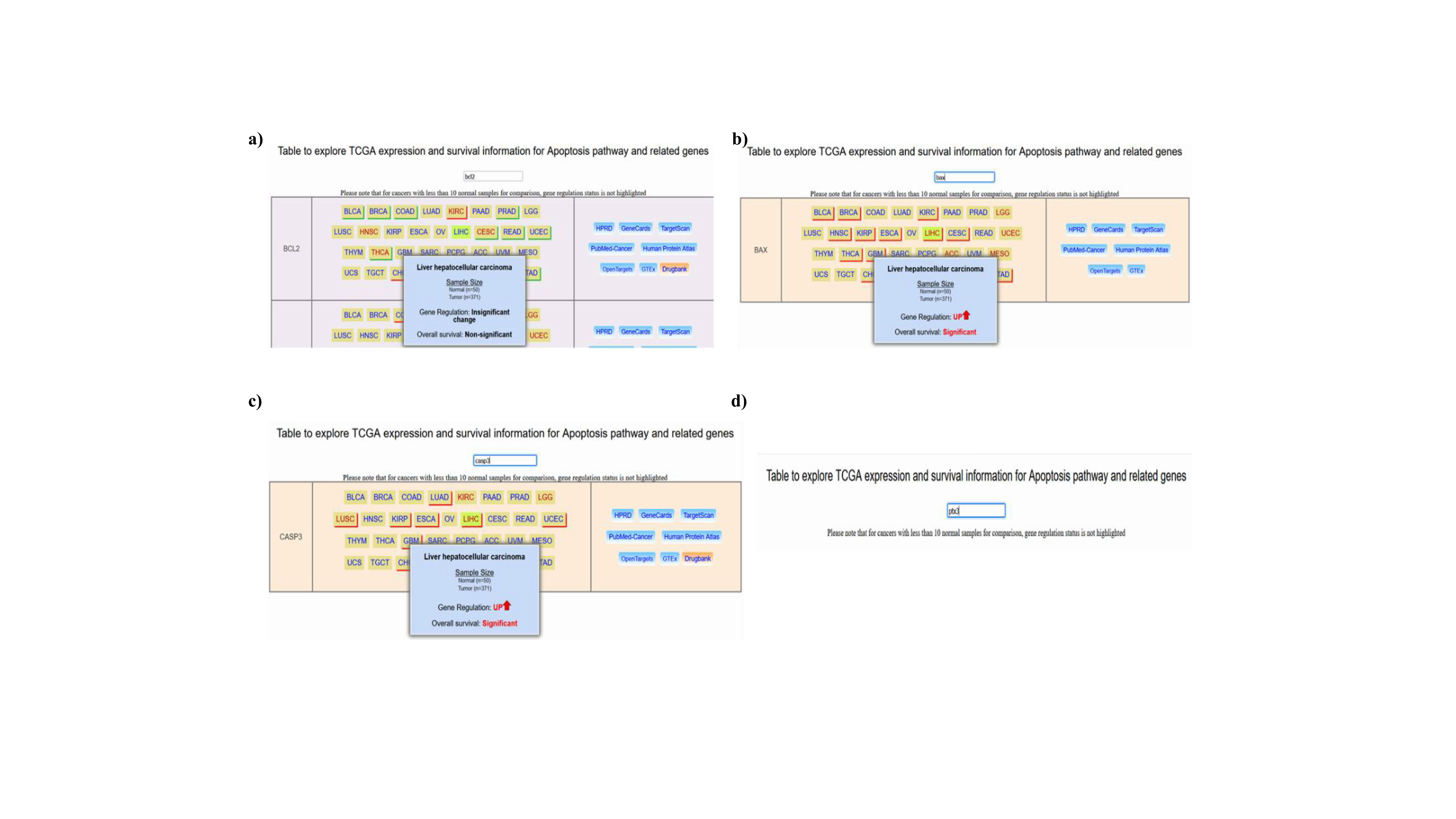

Supplement: Supplementary file 2 — Supplementary file2 Fig. 2 involvement of BAX, Bcl-2, CASP-3 and PTX3 genes during the apoptotic process with UALCAN data-web. Table to explore TGCA database expression data and survival information for apoptotic pathway and related genes: a) Bcl-2, b) BAX c) CASP-3 and d) PTX3 (PNG 1994 kb) [file 10585_2022_10164_MOESM2_ESM.png]
